# Fear of Death in Medical Students from a Peruvian University during the COVID-19 Pandemic

**DOI:** 10.3390/bs12050142

**Published:** 2022-05-13

**Authors:** Brayan Miranda-Chavez, Cesar Copaja-Corzo, Marco Rivarola-Hidalgo, Álvaro Taype-Rondan

**Affiliations:** 1 Facultad de Ciencias de la Salud, Universidad Privada de Tacna, Tacna 23003, Peru; brayanmch19@gmail.com (B.M.-C.); macrivarola@upt.pe (M.R.-H.); 2 Unidad de Investigación para la Generación y Síntesis de Evidencias en Salud, Universidad San Ignacio de Loyola, Lima 15024, Peru; alvaro.taype.r@gmail.com

**Keywords:** fear, death, education, medical, necrophobia, COVID-19, Collet–Lester scale, Peru

## Abstract

Due to close contact with death, medical students may question their own and their patients’ dying process, especially with the impact of the COVID-19 pandemic on the increase in deaths. This situation provokes fear and negative attitudes towards dealing with patients and their environment. This study aimed to assess the level of fear of death and associated factors in medical students at a Peruvian university. A cross-sectional analytical study was conducted during March 2021 in human medicine students from the first to the seventh year. A validated survey including the Collet–Lester fear-of-death scale was applied. Factors associated with the fear-of-death score were evaluated by calculating linear regression coefficients (β). A total of 284 students were included. The median age was 22 years, and 58.1% were female. The mean Collet–Lester scale score was 2.79, and it was higher in the dimensions related to the death of others. Adjusted analysis showed that the score on this scale was lower in students aged 24–40 years compared to 17–21 years (β: −0.25; 95% CI: −0.46 to −0.04) and those who had no religious beliefs (β: −0.29; 95% CI: −0.53 to −0.04). In conclusion, fear of death was lower than reported in other investigations despite the COVID-19 situation, being much lower among older students and those without religious beliefs.

## 1. Introduction

Fear of death has no clear definition, and while some authors interpret it as anxiety about death [1], others describe it as “an emotional reaction to the perception of signals involving subjective feelings of discomfort and worry, based on the contemplation or anticipation of various facets related to death, as well as the fact that death will be terrifying” [2,3,4].

One of the professions that has the most contact with death is medicine. Dealing with the process of death, medical students are frequently exposed during their internships, and it is to be expected that they will begin to question the dying process of themselves or their loved ones, which can cause fear and anguish, which if not adequately addressed could cause the student to distance him/herself from the scene [5,6], to acquire habits of distancing from the patient [7], reaching the therapeutic confinement of dying patients [8].

In medical students, fear of death has been addressed by a limited number of studies between the years 1998 and 2015 in Spain [9,10], Nigeria [11], the United States [12,13], the United Kingdom [5], Venezuela [14], and Hungary [15], and possible ways of addressing the conflict have been investigated [16]. In addition, due to the COVID-19 pandemic, the increase in deaths has been high, especially in Peru, which was the country with the highest mortality rate in the world [17]. This could have increased this fear in students of medicine or modified the way they process it. Despite this, no studies have been carried out in this context. Therefore, this study aimed to describe the fear of death and its associated factors in medical students at a university in Peru during the COVID-19 pandemic.

## 2. Materials and Methods

### 2.1. Design and Population

A cross-sectional study was carried out in March 2021 after one year of the appearance of the first cases of COVID-19 in Peru and with more than 40,000 deaths by that date [17]. The study was conducted at the Faculty of Health Sciences of the Private University of Tacna. This faculty is of private capital, began its functions in 1993, and is currently one of the two medical schools of Tacna, located in the south of Peru. Medical training in Peru lasts seven years, with the seventh year of study being known as a medical internship.

Medical students over 17 years of age who signed the informed consent form were included. Those with a diagnosis of any mental pathology such as depression, bipolar disorder, schizophrenia, and students who were not enrolled in any academic semester during the survey application were excluded from the study.

### 2.2. Procedures

After the study was approved, the delegates of each student year were asked for the complete list of students based on the course with the highest number of credits. These constituted the most significant number of students and were prerequisites for moving on to the following year. Subsequently, the researchers contacted students from each year of the course who were willing to collaborate in the study. They were trained using a virtual chat that lasted approximately 45 min, and they were instructed to survey each of the students in the year to which they had been assigned.

The collaborators were trained to approach each student using a private message through the WhatsApp application, accessing the pre-formed groups that the students had in that application. Subsequently, they had to send a brief message, which explained the informed consent and the survey to be filled out in the first instance. If the student agreed to participate in the study, they would be sent the link to the virtual survey in Google Forms. They collaborators were also advised to ask each student within two days if they had already completed the survey.

### 2.3. Instrument and Variables

The data collection instrument was a virtual survey in Google Forms. The primary variable included was fear of death, evaluated using the Collet–Lester scale created in 1969 [18], which was validated in Spanish in 2007 using nursing professionals and students in Spain, where it obtained a Cronbach’s alpha of 0.72 [19].

The Collet–Lester scale consists of 28 items distributed in four dimensions (7 items in each): “fear of one’s death”, “fear of one’s own dying process”, “fear of the death of others”, and “fear of the dying process of others”. The answers to each question have a Likert-type format, from 1 (not at all) to 5 (much). Finally, the average scores are presented, as well as the categories that are classified into: low fear of death (0 to 1), moderate fear of death (>1 to <4), and high fear of death (4 to 5) [20].

In addition, the following independent variables were included: age, gender, place of birth, marital status, number of children, religious belief, and academic year.

### 2.4. Ethical Aspects

This research followed the international guidelines of the Declaration of Helsinki, which provides the necessary procedures for research ethics [21]. The research ethics committee approved the study of the Faculty of Health Sciences of the Universidad Privada de Tacna (identification code: 050-FACSA-UI, 24 March 2021). Participation in the study was voluntary, in addition to prior acceptance of informed consent online. Likewise, the principles of autonomy, fairness, and confidentiality were respected.

### 2.5. Statistical Analysis

The database was downloaded in a Microsoft Excel document and exported for analysis to the statistical program STATA version 14 (StataCorp., College Station, TX, USA), where data analysis was performed. The descriptive statistics used were measures of central tendency and dispersion (numerical variables) or absolute frequencies and percentages (categorical variables).

In order to evaluate the factors associated with the Collet–Lester fear-of-death scale, beta coefficients and their respective 95% confidence intervals (95% CI) were calculated using linear regressions. In addition, those variables that obtained a *p* < 0.20 in the crude model were taken to elaborate the adjusted model.

## 3. Results

Of 461 medical students enrolled, 287 agreed to participate in the study, of which three were excluded because they did not complete the survey, so 284 students (61.6% of those enrolled) were finally included. The number of students enrolled by year of study is shown in Supplementary Materials (Table S1).

Of the 284 study participants, the median age was 22 years, 58.1% were female, 97.5% reported having no children, 51.8% were in fourth to seventh grade, 79.2% were from Tacna, and 82.2% professed some religious faith (Table 1).

Concerning the fear of death evaluated with the Collet–Lester scale, 93.66% had a moderate level of fear of dying, and the mean score was 2.79. The dimension with the highest mean score was the death of others (3.29), followed by the dying process of others (2.93), your dying process (2.66), and your death (2.29).

Within the dimension of the death of others, the items with the highest scores were those referring to the loss (3.90) and not being able to communicate with the loved one (3.72). In the dimension of the dying process, they saw their suffering (3.40) and did not know how to manage their grief at the loss of this person (3.22). In the dimension of your dying process were loss of faculties (2.94) and mental degeneration (2.92), and in terms of your death were dying young (2.79) and shortness of life (2.58) (Table 2).

When evaluating the factors associated with the fear-of-death scale score, it was found that students aged 24 to 40 years had lower fear-of-death scores than students aged 17 to 21 years (adjusted β: 0.25 points, 95% CI: −0.46 to −0.04), which was statistically significant for the dimensions of “in relation to one’s death” (adjusted β: −0.27, 95% CI: −0.50 to −0.03) and “in relation to the death of others” (adjusted β: −0.31, 95% CI: −0.58 to −0.04).

Likewise, those who reported having any religious beliefs had higher fear-of-death scores (adjusted β: 0.30, 95% CI: 0.05 to 0.50), which was statistically significant only for the “in relation to one’s death” dimension (adjusted β: −0.31, 95% CI: −0.59 to −0.03) (Table 3).

## 4. Discussion

### 4.1. Preponderant Dimension

The highest score (highest fear) was observed in the dimension of fear of the death of others, followed by the process of the death of others. This order coincides with other studies that applied the Collet–Lester scale to 25 medical students in the United States between 1989–1990 [12] and 444 nursing students in Spain in 2002 [22]. Other studies between 2009 and 2016 in medical or other health professions students also found that the highest-scoring dimension was fear of death of others, although the second-highest scoring dimension varied between studies [3,5,9,14,23]. This scenario may be in this way because, as other authors have suggested, Western societies currently prioritize the importance of another person’s death over their own [3,5,23]. After all, the latter is still considered taboo [10]. Although no pre–post analysis was performed in our study, this consistency with most studies suggests that the COVID-19 pandemic has not changed the prevalence of others’ fear of death over one’s own.

Finally, two other studies found higher scores on other dimensions: fear of the dying process of others in 175 medical students in Nigeria [11] and fear about one’s death in 676 medical students in Spain during 1998–1999 and 2013–2014 [10]. This suggests that fear of dying may vary according to the context, possibly due to beliefs about life and death or previous experiences in this regard, aspects that should be evaluated in future studies.

### 4.2. Scores

Our results indicate that the average score on the Collet–Lester scale was 2.79; this score is lower than the scores obtained in studies conducted in medical students in the countries of Spain (one study found 2.93 and another 3.50) [9,10], Nigeria (3.00) [11], the United Kingdom (3.22) [5], and Venezuela (3.60) [14]. It is not easy to compare these figures due to the different contexts in which each study was developed. Still, the hypothesis should be considered that despite more significant exposure to death, as has been happening in the COVID-19 pandemic, fear of death has not increased among medical students. Perhaps this is due to a rationalization of death [24].

Having a low level of fear in other people’s dying dimensions could be a sign of disinterest in patients’ treatment [5]. In our study, the percentage of students with a low level of fear of other people’s death and other people’s dying process was 1.8% and 3.2%, respectively, suggesting no marked disinterest in this regard.

On the other hand, a high level of fear of death could generate a negative attitude with distancing from the scene of the events [5] and an attitude in favor of prolonging the life of patients who will die anyway, leading to poor quality of life for patients and unnecessary expenses and anguish for family members [25]. However, these results are correlational, which does not imply causality, so each scenario must be evaluated in a particular way. In our study, the percentage of students with a high level of fear of other people’s death and the dying process of others was found to be 27.5% and 13.7%, respectively. In these dimensions, the items with the highest scores are those that associate this fear with the loss of a loved one, which could indicate a shortage of tools to cope with the grieving process.

### 4.3. Associated Factors

The older students presented lower scores on the fear-of-death scale, especially in its dimensions about their death and the death of others. This finding is similar to a study in social-health students in the years 2015–2016 [23] and to a study in nursing students in the years 2009–2010 [26], while another study in medical students with a 15-year interval (1998–1999; 2013–2014) reported that the older the students were, the more afraid of death, the death of others, and the dying process of others they were [10]. This contradiction may be because more than 70% of the participants were collected between 1998 and 2004, whereas the other studies (and our own) were conducted several years later, so temporal changes in thinking may have occurred.

Students who did not report having a religious belief obtained lower fear-of-death scores, especially on the dimension of self-death. This result contradicts precedents that have evaluated this association, which either finds that religious belief is associated with a lower level of fear of death [10,27,28] or does not find an association [3]. However, a systematic review found that fear of death is low in very religious individuals or those who do not have a religious belief (e.g., agnostics or atheists) [29]. This could be because fear of death makes people more likely to affiliate with a religious belief to shield themselves from fear of the dying process and death. It is also possible that a group of non-believers is currently rationalizing or adopting a secular view that accepts death as part of life, such as nihilistic optimism [28], which may reduce their fear of death, a hypothesis that merits further study.

No association was found between gender and fear of death. Other studies have found contradictory results because, while some find no differences [5,30,31], others find a greater fear of death in women [3,9,23]. Nor was an association found between years of study and fear of death. This is probably because, in the second year of the course, an anatomy course is taken, in which students are in contact with natural human bodies and perform dissections, which may initiate early contact with death with these reflections.

There were some limitations in the present study. First, due to the COVID-19 pandemic, the survey was conducted online, which could have influenced the data extraction somewhat. We were also unable to assess the impact of the COVID-19 pandemic on fear of death because we did not carry out a pre-and-post-pandemic analysis. Second, the study is cross-sectional, so it will not be possible to establish a follow-up on students’ attitudes or to explore causality. Third, it is possible to have residual confounding due to adjustment for limited covariates. Finally, our research has not considered the students’ personality traits, which could have influenced their responses during the study.

## 5. Conclusions

In conclusion, the score obtained on the fear-of-death scale was lower than in previous studies on medical students, despite the COVID-19 situation. The dimension of fear of death with the highest score was the fear of death of other people. Likewise, a higher level of fear was evidenced among the youngest and those with religious beliefs, unlike those reported in other studies; these associations should be confirmed and explored in future studies.

## Figures and Tables

**Table 1:** Characteristics of participants (*n* = 284).

Features	*n* (%)
Age in years	22 (20–24) *
Age in years by tertiles:	
17 to 21	127 (44.7)
22 to 23	75 (26.4)
24 to 40	82 (28.8)
Female gender	165 (58.1)
Marital status single	282 (99.3)
Has at least one child	7 (2.5)
Year of study:	
Basic sciences (first to the third year)	137 (48.2)
Clinics (fourth to the seventh year)	147 (51.8)
Place of birth:	
Tacna	225 (79.2)
Arequipa	22 (7.7)
Puno	9 (3.2)
Moquegua	6 (3.1)
Others	22 (7.5)
If you have any religious beliefs	242 (85.2)

* Median and interquartile range.

**Table 2:** Collet–Lester fear-of-death scale.

How Concerned or Anxious Are You about the Following Aspects of Death and the Dying Process?	Score: Mean (Standard Deviation)	Categories		
		Under (Average Score from 0 to 1) N (%)	Moderate (Average Score from >1 to <4) N (%)	High (Average Score from 4 to 5) N (%)
Dimension 1: In relation to one’s own death	2.29 (0.86)	19 (6.7)	256 (90.1)	9 (3.2)
1. Total loneliness at death	2.16 (1.11)	94 (33.1)	153 (53.9)	37 (13.0)
2. The brevity of life	2.58 (1.16)	61 (21.5)	160 (56.3)	63 (22.2)
3. All the things you will lose when you die	2.03 (1.18)	128 (45.1)	119 (41.9)	37 (13.0)
4. Dying young	2.79 (1.25)	51 (18.0)	151 (53.2)	82 (28.9)
5. What it will be like to be dead	2.36 (1.28)	91 (32.0)	137 (48.2)	56 (19.7)
6. Not being able to think or experience anything anymore	2.46 (1.27)	81 (28.5)	142 (50.0)	61 (21.5)
7. The disintegration of the body after death	1.61 (0.91)	172 (60.6)	96 (33.8)	16 (5.6)
Dimension 2: About your own dying process.	2.66 (0.91)	14 (4.9)	243 (85.6)	27 (9.5)
1. The physical degeneration involved in the process of dying	1.91 (1.02)	123 (43.3)	138 (48.6)	23 (8.1)
2. The pain involved in the dying process	2.72 (1.16)	45 (15.9)	170 (59.9)	69 (24.3)
3. The mental degeneration of aging	2.92 (1.20)	36 (12.7)	154 (54.2)	94 (33.1)
4. Loss of faculties during the dying process	2.94 (1.11)	28 (9.9)	163 (57.4)	93 (32.8)
5. Uncertainty about how bravely you will face the dying process	2.58 (1.16)	53 (18.7)	177 (62.3)	54 (19.0)
6. Your lack of control over the dying process	2.63 (1.24)	58 (20.4)	151 (53.2)	75 (26.4)
7. The prospect of dying in a hospital far from friends and family	2.89 (1.30)	47 (16.6)	143 (50.4)	94 (33.1)
Dimension 3: In relation to the death of others	3.29 (0.98)	5 (1.8)	201 (70.8)	78 (27.5)
1. The loss of a loved one	3.90 (1.10)	8 (2.8)	87 (30.6)	189 (66.6)
2. Having to see his corpse	3.03 (1.29)	36 (12.7)	144 (50.7)	104 (36.6)
3. Not being able to communicate with her anymore	3.72 (1.24)	15 (5.3)	98 (34.5)	171 (60.2)
4. Regret did not get along better with her when she was still alive.	3.30 (1.26)	26 (9.2)	133 (47.0)	124 (43.8)
5. Growing old alone, without a loved one	3.25 (1.27)	26 (9.2)	133 (46.8)	125 (44.0)
6. Feeling guilty about the relief caused by your death	2.46 (1.22)	74 (26.1)	149 (52.5)	61 (21.5)
7. Feeling lonely without her	3.32 (1.26)	23 (8.1)	121 (42.6)	140 (49.3)
Dimension 4: In relation to the dying process of others.	2.93 (0.91)	9 (3.2)	236 (83.1)	39 (13.7)
1. Having to be with someone who is dying	2.95 (1.16)	29 (10.2)	161 (56.7)	94 (33.1)
2. Having to be with someone who wants to talk about death with you	2.16 (1.03)	90 (31.7)	169 (59.5)	25 (8.8)
3. See how he suffers pain	3.40 (1.18)	17 (6.0)	136 (47.9)	131 (46.1)
4. Observing the physical degeneration of your body	2.91 (1.09)	28 (9.7)	178 (62.7)	78 (27.5)
5. Not knowing how to manage your grief in the face of the loss of a loved one	3.22 (1.22)	21 (7.4)	148 (52.1)	115 (40.5)
6. Assist in the deterioration of their mental faculties	3.11 (1.13)	22 (7.8)	156 (54.9)	106 (37.3)
7. To be aware that someday you will also live this experience	2.73 (1.21)	51 (18.0)	156 (54.9)	77 (27.1)
Total	2.79 (0.76)	2 (0.7)	265 (93.3)	17 (6.0)

**Table 3:** Factors associated with the Collet–Lester fear-of-death scale score in the study population.

Variable	Total		Dimension 1		Dimension 2		Dimension 3		Dimension 4	
	Crude β (95% CI)	Adjusted β (95% CI)	Crude β (95% CI)	Adjusted β (95% CI)	Crude β (95% CI)	Adjusted β (95% CI)	Crude β (95% CI)	Adjusted β (95% CI)	Crude β (95% CI)	Adjusted β (95% CI)
Age (years)										
17 to 21	Ref	Ref	Ref	Ref	Ref	Ref	Ref	Ref	Ref	Ref
22 to 23	−0.06 (−0.28 to 0.15)	−0.04 (−0.26 to 0.18)	0.07 (−0.17 to 0.32)	0.10 (−0.14 to 0.34)	−0.13 (−0.39 to 0.14)	-	−0.06 (−0.34 to 0.22)	−0.02 (−0.30 to 0.26)	−0.14 (−0.40 to 0.11)	−0.01 (−0.31 to 0.29)
24 to 40	−0.27 (−0.48 to −0.06)	−0.25 (−0.46 to −0.04)	−0.28 (−0.52 to −0.05)	−0.27 (−0.50 to −0.03)	−0.18 (−0.43 to 0.07)	-	−0.32 (−0.60 to −0.05)	−0.31 (−0.58 to −0.04)	−0.28 (−0.53 to −0.03)	−0.16 (−0.46 to 0.14)
Sex										
Female	Ref	-	Ref	-	Ref	Ref	Ref	Ref	Ref	Ref
Male	−0.10 (−0.28 to 0.08)	-	0.06 (−0.14 to 0.26)	-	0.03 (−0.18 to 0.25)	-	−0.26 (−0.49 to −0.03)	−0.23 (−0.46 to 0.00)	−0.22 (−0.43 to −0.01)	−0.19 (−0.40 to 0.03)
Children										
No children	Ref	-	Ref	-	Ref	Ref	Ref	Ref	Ref	Ref
With children	−0.18 (−0.76 to 0.39)	-	−0.05 (−0.69 to 0.60)	-	−0.07 (−0.76 to 0.62)	-	−0.42 (−1.15 to 0.32)	-	−0.20 (−0.89 to 0.48)	-
Academic year										
1st to 3rd	Ref	-	Ref	-	Ref	Ref	Ref	Ref	Ref	Ref
4th to 7th	−0.11 (−0.29 to 0.07)	-	0.01 (−0.19 to 0.21)	-	−0.14 (−0.35 to 0.71)	−0.13 (−0.35 to 0.08)	−0.05 (−0.28 to 0.17)	-	−0.25 (−0.46 to −0.04)	−0.18 (−0.44 to 0.09)
Religious belief										
Yes	Ref	Ref	Ref	Ref	Ref	Ref	Ref	Ref	Ref	Ref
No	−0.30 (−0.50 to −0.05)	−0.29 (−0.53 to −0.04)	−0.30 (−0.58 to −0.02)	−0.31 (−0.59 to −0.03)	−0.23 (−0.53 to 0.07)	−0.21 (−0.51 to 0.08)	−0.36 (−0.68 to −0.04)	−0.29 (−0.62 to 0.03)	−0.29 (−0.59 to 0.00)	−0.22 (−0.52 to 0.08)

## Data Availability

Data collected and analyzed during the study are available upon reasonable request.

## Supplementary Materials

The following supporting information can be downloaded at: https://www.mdpi.com/article/10.3390/bs12050142/s1, Table S1: The number of students enrolled by year of study.
