# Supplementary material for: Fear of Death in Medical Students from a Peruvian University during the COVID-19 Pandemic
Source: Behav Sci (Basel). 2022 May 13;12(5):142. doi: 10.3390/bs12050142 (PMC9137769; doi:10.3390/bs12050142)
Supplement: Supplementary file 1 [file behavsci-12-00142-s001.zip › behavsci-1675030-supplementary.pdf]

**Supplementary Materials**

**Table S1.** The number of students enrolled by year of study.

| Year of study | Number of students enrolled | Number of students included in the study *n* (%) |
|---|---|---|
| First year | 90 | 45 (50.0) |
| Second year | 86 | 46 (53.5) |
| Third year | 52 | 46 (88.5) |
| Fourth year | 59 | 39 (66.1) |
| Fifth year | 55 | 37 (67.3) |
| Sixth year | 66 | 47 (71.2) |
| Seventh year | 53 | 24 (45.3) |
| Total | 461 | 284 (61.6) |
